# Temporal relationships between maternal metabolic parameters with neonatal adiposity in women with obesity differ by neonatal sex: Secondary analysis of the DALI study

**DOI:** 10.1111/ijpo.12628

**Published:** 2020-03-06

**Authors:** Rodrigo A. Lima, Gernot Desoye, David Simmons, Roland Devlieger, Sander Galjaard, Rosa Corcoy, Juan M. Adelantado, Fidelma Dunne, Jürgen Harreiter, Alexandra Kautzky‐Willer, Peter Damm, Elisabeth R. Mathiesen, Dorte M. Jensen, Lise‐Lotte Andersen, Mette Tanvig, Annunziata Lapolla, Maria G. Dalfra, Alessandra Bertolotto, Ewa Wender‐Ozegowska, Agnieszka Zawiejska, David J. Hill, Frank J. Snoek, Judith G. M. Jelsma, Mireille N. M. van Poppel

**Affiliations:** ^1^ Institute of Sport Science University of Graz Graz Austria; ^2^ Department of Obstetrics and Gynecology Medizinische Universitaet Graz Graz Austria; ^3^ Western Sydney University Campbelltown New South Wales Australia; ^4^ The Institute of Metabolic Science Addenbrooke's Hospital Cambridge UK; ^5^ KU Leuven Department of Development and Regeneration: Pregnancy, Fetus and Neonate, Gynaecology and Obstetrics University Hospitals Leuven Leuven Belgium; ^6^ Department of Obstetrics and Gynaecology, Division of Obstetrics and Prenatal Medicine, Erasmus MC University Medical Centre Rotterdam The Netherlands; ^7^ Institut de Recerca de l'Hospital de la Santa Creu i Sant Pau Barcelona Spain; ^8^ CIBER Bioengineering, Biomaterials and Nanotechnology Instituto de Salud Carlos III Zaragoza Spain; ^9^ Galway Diabetes Research Centre and College of Medicine Nursing and Health Sciences National University of Ireland Galway Ireland; ^10^ Gender Medicine Unit, Endocrinology and Metabolism, Department of Internal Medicine III Medical University of Vienna Vienna Austria; ^11^ Center for Pregnant Women with Diabetes, Department of Endocrinology and Obstetrics, Rigshospitalet Institute of Clinical Medicine, Faculty of Health and Medical Sciences, University of Copenhagen Copenhagen Denmark; ^12^ Steno Diabetes Center Odense Odense University Hospital Odense Denmark; ^13^ Department of Gynecology and Obstetrics Odense University Hospital Odense Denmark; ^14^ Department of Clinical Research, Faculty of Health Sciences University of Southern Denmark Odense Denmark; ^15^ Universita Degli Studi di Padova Padua Italy; ^16^ Azienda Ospedaliero Universitaria – Pisa Pisa Italy; ^17^ Medical Faculty I Poznan University of Medical Sciences Poznań Poland; ^18^ Recherche en Santé Lawson SA Bronschhofen Switzerland; ^19^ Department of Medical Psychology Amsterdam UMC, Vrije Universiteit Amsterdam, Amsterdam Public Health Research Institute Amsterdam The Netherlands; ^20^ Department of Public and Occupational Health Amsterdam UMC, Vrije Universiteit Amsterdam, Amsterdam Public Health research institute Amsterdam The Netherlands

**Keywords:** foetal growth, foetal programming, maternal health, metabolic syndrome, neonatal body composition, pregnancy

## Abstract

**Objectives:**

To investigate the importance of time in pregnancy and neonatal sex on the association between maternal metabolic parameters and neonatal sum of skinfolds.

**Methods:**

This was a longitudinal, secondary analysis of the vitamin D and lifestyle intervention for gestational diabetes mellitus study, conducted in nine European countries during 2012 to 2015. Pregnant women with a pre‐pregnancy body mass index (BMI) of ≥29 kg/m^2^ were invited to participate. We measured 14 maternal metabolic parameters at three times during pregnancy: <20 weeks, 24 to 28 weeks, and 35 to 37 weeks of gestation. The sum of four skinfolds assessed within 2 days after birth was the measure of neonatal adiposity.

**Results:**

In total, 458 mother‐infant pairs (50.2% female infants) were included. Insulin resistance (fasting insulin and HOMA‐index of insulin resistance) in early pregnancy was an important predictor for boys' sum of skinfolds, in addition to fasting glucose and maternal adiposity (leptin, BMI and neck circumference) throughout pregnancy. In girls, maternal lipids (triglycerides and fatty acids) in the first half of pregnancy were important predictors of sum of skinfolds, as well as fasting glucose in the second half of pregnancy.

**Conclusions:**

Associations between maternal metabolic parameters and neonatal adiposity vary between different periods during pregnancy. This time‐dependency is different between sexes, suggesting different growth strategies.

**Abbreviations**
BMI: body mass index
DALI: vitamin D and lifestyle intervention for gestational diabetes mellitus prevention: an European multicentre randomized trial
GDM: gestational diabetes
HAPO: hyperglycemia and adverse pregnancy outcome
HOMA‐IR: HOMA‐index of insulin resistance

## 1 INTRODUCTION

Globally, in 2017 an estimated 38 million children under 5 years of age presented either overweight or obesity. This is a major public health concern.^1^ Hence, strategies have to be developed to reduce this burden and prevent childhood obesity. This requires a thorough identification and understanding of the underlying determinants.

Maternal obesity in pregnancy is linked with the development of neonatal and childhood adiposity.^2^, ^3^, ^4^, ^5^ Although there is much interest in the relationship between maternal obesity and childhood adiposity, maternal metabolic factors driving the increase in offspring adiposity have not been fully understood.

The Hyperglycemia and Adverse Pregnancy Outcome study showed a continuous positive relationship of maternal glucose in the second trimester with measures of neonatal body fat.^6^ Findings from the Healthy Start Study support this and, in addition, showed temporal changes in the association of glucose and neonatal fat, with stronger associations in the second half of pregnancy compared to early pregnancy.^7^, ^8^ However, other studies clearly indicate that the influence of maternal phenotype on foetal growth can already be demonstrated in the early pregnancy period: foetal abdominal circumference, as proxy for foetal overgrowth, is increased in women with obesity and/or gestational diabetes (GDM) already at 20 weeks of gestation or even before^9^, ^10^; maternal insulin resistance in the first half of pregnancy is related to neonatal fat percentage,^7^ and fasting glucose in the first trimester with the risk of a large‐for‐gestational age baby.^11^ Although these studies suggest an influence of the early pregnancy period, surprisingly little is known about temporal relations between maternal metabolic measures and neonatal fat accrual.^12^

Female neonates are known to be more insulin resistant,^13^, ^14^ which might explain why males are more affected by in utero exposure to gestational diabetes.^9^ Therefore, it is mandatory to assess temporal relations of maternal metabolism with neonatal adiposity in a sex‐dependent manner.

In this study, we investigated the association between maternal health measures at different times during pregnancy and neonatal sum of skinfolds in a sex‐specific manner. We hypothesized that: (a) associations between maternal metabolic parameters and neonatal adiposity differ at different time points, and (b) these temporal differences are sex‐specific. We tested these hypotheses in 458 mother‐infant pairs in the vitamin D and lifestyle intervention for gestational diabetes mellitus (DALI) study, a pan‐European study originally designed for the prevention of gestational diabetes.^15^

## 2 METHODS

### 2.1 Design and participants

This is a longitudinal, secondary analysis of the DALI study, which was a multicentre parallel randomized trial conducted in nine European countries (Austria, Belgium, Denmark, Ireland, Italy, Netherlands, Poland, Spain and United Kingdom) during 2012 to 2015. The study was prospectively registered as a randomized clinical trial (RCT) with the primary aim to prevent gestational diabetes mellitus on November 21, 2011 (ISRCTN70595832). Local ethics committee approval and written informed consent of all women was obtained. Pregnant women with a pre‐pregnancy body mass index (BMI) of ≥29 kg/m^2^, <20 weeks of gestation, a singleton pregnancy and aged ≥18 years were invited to participate.^16^ Exclusion criteria included diagnosis with early gestational diabetes mellitus,^17^, ^18^ pre‐existing diabetes, and chronic medical conditions.

### 2.2 Design and procedures

The study was originally designed as an RCT with the following groups, pre‐stratified for site: (a) healthy eating; (b) physical activity; (c) healthy eating + physical activity; (d) healthy eating + physical activity + vitamin D; (e) healthy eating + physical activity + placebo; (f) vitamin D; (g) placebo, (h) control. Staff involved with measurements, but not participants, were blinded to the lifestyle intervention. Both staff and participants were blinded to vitamin D intervention. For the purpose of this analysis, the data were analyzed as a longitudinal cohort. Maternal measurements took place at baseline (<20 weeks), at 24 to 28 and at 35 to 37 weeks of gestation. Since methodology has been extensively described elsewhere,^16^ only variables of interest will be detailed in this manuscript.

### 2.3 Measurements

#### 2.3.1 Neonatal outcome

Triceps, subscapular, supra‐iliac and quadriceps skinfolds were measured within 48 hours of birth with a Harpenden skinfold calliper (Baty, UK), and the values summed to obtain the primary neonatal outcome measure, the sum of skinfolds. Each skinfold measurement was measured twice and if a difference of more than 0.2 mm was registered, a third measurement was performed and the average of the three was taken. The neonatal age at the measurement was the time between birth and measurements, which was registered in hours.

#### 2.3.2 Maternal metabolic and adiposity parameters

Maternal height was determined at baseline with a stadiometer (SECA 206; SECA, UK). Women were weighed on calibrated electronic scales (SECA 888 and 877, SECA, UK) at baseline (<20 weeks), 24 to 28 weeks, and at 35 to 37 weeks of gestation. BMI was calculated as weight in kilogram divided by the square of height in metres. Gestational weight gain was defined as the change in objectively measured weight from pre‐pregnancy to <20, <20 to 24‐28 weeks and from 24‐28 to 35‐37 weeks. Neck circumference was obtained in a standing relaxed upright position between mid‐cervical spine and mid‐anterior neck, to within 1 mm.^19^

After fasting for 10 hours, blood was collected and mothers consumed a 250 mL 75 g glucose drink (within a period of 5 minutes). Further blood collections took place after 60 and 120 minutes. All the samples were centrifuged and separated aliquots of plasma (1000 μL or 250 μL) placed in microrack tubes and stored at −20°C or −80°C in the central trial laboratory, prior to analysis, in Graz, Austria. The maternal concentrations of plasma fasting glucose, fasting insulin, triglycerides, free fatty acids and leptin were quantified. For insulin and leptin commercially available Enzyme‐Linked Immuno Sorbent Assays were used. In the samples taken 60 and 120 minutes after glucose load, only glucose (1‐hour glucose and 2‐hour glucose) and insulin (1‐hour insulin and 2‐hour insulin) were assessed. Fasting insulin resistance was derived from homeostasis model assessment (HOMA‐index of insulin resistance – HOMA‐IR).^20^ Insulin secretion after glucose load were calculated with the Stumvoll first phase and Stumvoll second phase validated equations.^21^

#### 2.3.3 Covariates

Information on possible covariates was collected in the baseline questionnaire or from medical files: national site(s) of recruitment, maternal age, gestational age during pregnancy (<20, 24 to 28 and 35 to 37 weeks), maternal ethnicity (European or non‐European descent), maternal education (low, medium and high), smoking status at 35 to 37 weeks of gestation (yes/no), pre‐pregnancy BMI and gestational age at birth.

### 2.4 Statistical analyses

All analyses were performed in STATA version 13 for windows (StataCorp LP, College Station, Texas) and a 5% type I error rate was used for the analyses. We have 80% statistical power to detect associations with small effect sizes (*f* ^2^ = 0.04) considering the number of participants evaluated (n = 458) and the number of exposures and confounding variables in each of our models. Skewed variables were log‐transformed before analyses (fasting insulin, 1‐hour insulin, 2‐hour insulin, HOMA‐IR, Stumvoll first phase, Stumvoll second phase, triglycerides, leptin and BMI). Independent *t* tests were performed to evaluate descriptive differences in the mother's metabolic and adiposity parameters for each pregnancy period in relation to their child's sex (results shown in Table 1). Independent *t* tests also evaluated sex‐differences in neonatal skinfolds.

**Table 1:** Mean and SD of the mothers' variables included in the main analyses for different gestational time and stratified by sex

Maternal parameters	<20 weeks					24‐28 weeks					35‐37 weeks				
	Boys		Girls		*P*	Boys		Girls		*P*	Boys		Girls		*P*
	Mean	SD	Mean	SD		Mean	SD	Mean	SD		Mean	SD	Mean	SD	
Fasting glucose (mmol/L)	4.58	0.38	4.61	0.36	.39	4.54	0.40	4.61	0.43	.07	4.47	0.45	4.56	0.43	**.05**
1‐h glucose (mmol/L)	6.60	1.38	6.80	1.47	.15	7.56	1.70	7.77	1.60	.19	7.93	1.55	8.12	1.45	.25
2‐h glucose (mmol/L)	5.72	1.11	5.81	1.18	.45	6.17	1.25	6.15	1.27	.87	6.44	1.25	6.49	1.12	.70
Fasting insulin (μU/mL)	12.69	1.03	13.12	1.03	.46	13.98	1.03	15.46	1.03	**.02**	17.05	1.04	17.97	1.04	.37
1‐h insulin (μU/mL)	85.48	1.05	84.98	1.05	.93	112.62	1.04	124.58	1.04	.09	157.47	1.05	166.11	1.05	.43
2‐h insulin (μU/mL)	60.87	1.05	53.62	1.06	.43	72.44	1.05	76.22	1.05	.47	107.99	1.06	110.62	1.06	.75
HOMA‐IR ([μU/mL]*[mmol/L])	2.57	1.03	2.68	1.03	.40	2.84	1.03	3.15	1.03	**.03**	3.31	1.04	3.62	1.05	.16
Stumvoll first phase (pmol/L)	1639.26	1.02	1616.57	1.02	.68	1740.14	1.02	1872.26	1.03	**.04**	2556.15	1.03	2676.00	1.03	.31
Stumvoll second phase (pmol/L)	421.81	1.02	416.57	1.02	.70	449.52	1.02	482.72	1.02	**.03**	650.25	1.03	680.23	1.03	.30
Triglycerides (mmol/L)	1.23	1.02	1.26	1.02	.48	1.76	1.02	1.70	1.02	.25	2.21	1.03	2.13	1.03	.33
Fatty acids (mmol/L)	0.63	0.23	0.64	0.21	.48	0.59	0.23	0.58	0.19	.37	0.59	0.23	0.61	0.21	.40
Leptin (pg/mL)	33.63	1.03	34.16	1.03	.74	32.59	1.03	34.29	1.04	.30	32.07	1.04	33.02	1.04	.59
Neck circumference (cm)	36.18	2.06	36.21	2.17	.89	36.29	2.12	36.28	2.23	.98	36.53	2.17	36.60	2.22	.74
BMI (kg/m^2^)	34.13	1.01	34.17	1.01	.91	35.63	1.01	35.64	1.01	.97	36.88	1.01	36.96	1.01	.84
Gestational weight gain (kg)	2.02	3.92	1.84	4.48	.64	4.06	3.28	3.92	2.74	.63	3.82	2.78	3.76	2.82	.82

*Note: P* value from independent *t* test; Fasting insulin, 1‐hour insulin, 2‐hour insulin, HOMA‐IR, Stumvoll first phase, Stumvoll second phase, triglycerides, leptin and BMI were log‐transformed for the analyses, but the values in the table were exponentiated for clarity.

Abbreviation: BMI, body mass index.

We conducted principal component factor analyses for each measurement time during pregnancy to group the exposures with shared variances in the same factor. All components from the mother's health profile were included in the factor analyses: fasting glucose, 1‐hour glucose, 2‐hour glucose, fasting insulin, 1‐hour insulin, 2‐hour insulin, HOMA‐IR, Stumvoll first phase, Stumvoll second phase, triglycerides, free fatty acids, leptin, neck circumference, BMI and gestational weight gain. Independent of the pregnancy period, the scree plot determined that four factors were relevant to be estimated. Thus, four factors were estimated for each time during pregnancy by the following criteria:

1. Independent of the time during pregnancy, each factor should be composed of the same variables;
2. Each factor was composed of variables with high loading factors (≥0.4) in at least two measuring points after varimax rotation of the correlation matrix;
3. Components with high loading factors (≥0.4) in more than one factor were only included in the factor with the highest loading factor;
4. Factors were estimated using the linear regression prediction score.

At all three time points during pregnancy, factor 1 was composed of fasting insulin and HOMA‐IR (representing insulin resistance); factor 2 was composed of 1‐hour insulin, 2‐hour insulin, Stumvoll first phase and Stumvoll second phase (representing insulin response); factor 3 was constituted of 1‐hour glucose and 2‐hour glucose (representing glucose tolerance), and, factor 4 was composed of leptin, BMI and neck circumference (representing maternal adiposity). Table S1 presents the cumulative variance of each factor in the three periods of gestation. Fasting glucose, triglycerides, free fatty acids and gestational weight gain did not have a high loading factor for any of the factors, nor did they built a separate factor, and were not included in any of the factors. Thus, they were analyzed as single exposures in relation to neonatal sum of skinfolds.

The association of each resulting factor from the mother's health profile with neonatal sum of skinfolds was assessed using linear multilevel analyses, adjusted for intervention group, site of recruitment, gestational age during pregnancy, gestational age at birth, neonatal age at measurement, maternal ethnicity, maternal education, BMI (except in factor 4 since BMI was part of this factor), maternal age, smoking status and the cluster structure. Sex‐effects were estimated by inclusion of an interaction term between the factor and sex. We used robust correction for estimating the standard errors and estimated *P*‐values using the Bonferroni adjustment for multiple testing. The association between the mother's health profile not included in any of the factors (fasting glucose, triglycerides, free fatty acids and gestational weight gain) and neonatal sum of skinfolds was evaluated following the same procedure abovementioned. Note that we tested the same analysis replacing BMI for gestational weight gain as a confounder. The conclusions were similar, so we only present the results with BMI adjustment.

### 2.5 Supplementary analysis

Multiple linear multilevel analyses tested the association between each of the maternal measurements during pregnancy and neonatal sum of skinfolds (see Table S2). Sex‐effects were estimated by inclusion of an interaction term between the exposures and sex. All analyses were adjusted for intervention group, site of recruitment, maternal ethnicity, education, BMI (except when BMI was the exposure), age, smoking status, gestational age during pregnancy (<20, 24 to 28 and 35 to 37 weeks), gestational age at birth (weeks) and neonatal age at measurement (hours post birth). The cluster structure of data was also taken into account with individuals nested into the site of recruitment. We used robust correction for estimating the standard errors and used the Bonferroni adjustment for multiple testing. Analyses with triglycerides and fatty acids as exposures were further adjusted for HOMA‐IR.

## 3 RESULTS

In total, 458 mother‐infant pairs (50.2% female infants) were included in the study. On average, the mothers were 32.1 (±5.3) years of age and their infants were measured 19.9 (±30.5) hours after birth. Mothers gave birth after 39.7 (±1.4) weeks of gestation, and most mothers perceived themselves as of European descent (84.6%) and did not smoke during pregnancy (85.6%). More than half of the mothers (57.6%) reported high educational level, whereas 32.0% reported medium educational level. Neonatal boys exhibited lower sum of skinfolds compared to girls (Mean sum of skinfolds_boys_ = 20.3 mm [±5.2]; Mean sum of skinfolds_girls_ = 21.5 mm [±5.4]; *P* = .02).

### 3.1 Maternal metabolic parameters at three time points

Table 1 describes the unadjusted sex‐differences in the mothers' metabolic profile for each gestational time. In the first measurement prior to 20 weeks of gestation, no differences were observed in maternal metabolic measurements between mothers of boys and girls. At 24 to 28 weeks of gestation, mothers of girls had higher fasting insulin, HOMA‐IR and Stumvoll first and second phases compared to mothers of boys. At 35 to 37 weeks of gestation, mothers of girls had higher fasting glucose compared to those of boys (Table 1).

### 3.2 Associations of maternal metabolic parameters with neonatal adiposity

**Figure 1:**
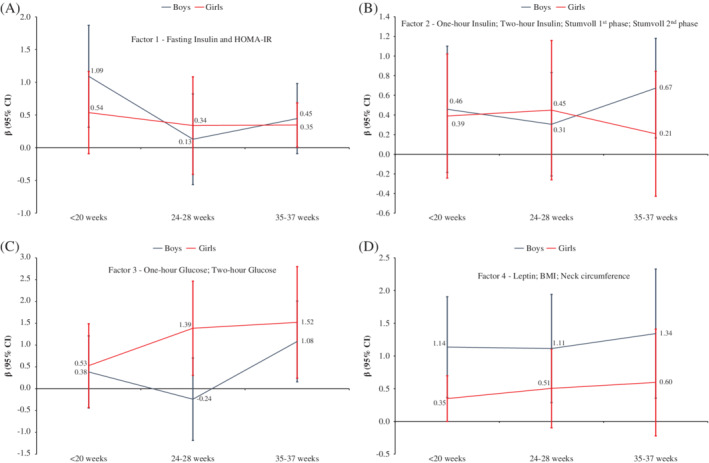
Multilevel regression coefficients of the association between (A) factor 1 (fasting insulin and HOMA‐index); (B) factor 2 (1‐hour insulin, 2‐hour insulin, Stumvoll phase 1 and Stumvoll phase 2); (C) factor 3 (1‐hour glucose and 2‐hour glucose) and (D) factor 4 (leptin, BMI and neck circumference) and neonatal sum of skinfolds (mm) by sex in three periods of gestation. Adjusted intervention group, site of recruitment, gestational age during pregnancy, gestational age at birth, neonatal age at measurement, maternal ethnicity, maternal education, body mass index (BMI), except in the factor 4 since BMI was part of this factor, maternal age, smoking status and cluster structure (individuals nested in site of measurement)

Associations of the four factors, and fasting glucose, triglycerides, fatty acids and gestational weight gain at the three time points with neonatal adiposity are described in Table 2. Factor 1 (fasting insulin and HOMA‐IR) at <20 weeks of gestation was associated with boys' sum of skinfolds, and with girls' sum of skinfolds at 35 to 37 weeks of gestation. Factor 2 (1‐hour insulin, 2‐hour insulin, Stumvoll first phase, Stumvoll second phase) was associated with boys' sum of skinfolds at 35 to 37 weeks of gestation, and no association was found of this factor with girls' sum of skinfolds. Factor 3 (1‐hour glucose and 2‐hour glucose) at 35 to 37 weeks of gestation was associated with boys' sum of skinfolds and at 24 to 28 and 35 to 37 weeks of gestation with girls' sum of skinfolds. Finally, factor 4 (leptin, BMI and neck circumference) was associated with boys' sum of skinfolds in all pregnancy periods, but not with girls' sum of skinfolds (Figure 1).

**Table 2:** Multilevel regression coefficients of the association between maternal health profile components and child's sum of skinfolds (mm) by sex in three periods of gestation

Maternal parameters	Boys						Girls					
	<20 weeks		24‐28 weeks		35‐37 weeks		<20 weeks		24‐28 weeks		35‐37 weeks	
	*β*	*P*	*β*	*P*	*β*	*P*	*β*	*P*	*β*	*P*	*β*	*P*
Factor 1 Fasting insulin and HOMA‐IR	1.091	**.006**	.130	.713	.445	.103	.536	.094	.339	.372	.347	**.044**
Factor 2 1‐h and 2‐h insulin, and Stumvoll first and second phases	.458	.163	.305	.256	.673	**.009**	.389	.228	.449	.215	.208	.521
Factor 3 1‐h and 2‐h glucose	.384	.362	−.241	.617	1.083	**.022**	.529	.280	1.387	**.012**	1.520	**.020**
Factor 4 Leptin, BMI and neck circumference	1.136	**.004**	1.114	**.008**	1.342	**.008**	.349	.196	.505	.100	.596	.152
Fasting glucose (mmol/L)	1.792	**<.001**	1.406	**.011**	2.085	**.019**	−.045	.968	2.475	**.008**	1.361	**.035**
Triglycerides (log)	1.428	.423	.235	.854	2.740	**.005**	2.005	**.031**	1.907	.119	1.395	.235
Fatty acids (mmol/L)	1.555	.152	.462	.787	1.179	.544	3.886	**<.001**	4.942	**.011**	−1.678	.203
Gestational weight gain (kg)	.197	**.033**	−.003	.975	.059	.645	.021	.646	.125	.345	.010	.526

*Note:* Adjusted for intervention group, site of recruitment, maternal ethnicity, education, BMI (except when BMI was the exposure), age and smoking status, gestational age during pregnancy (weeks), gestational age at birth (weeks) and neonatal age at measurement (hours), and cluster structure (individuals nested in site of measurement). Besides the aforementioned adjustments, HOMA‐index was also an adjustment when triglycerides and fatty acids were the exposures. *P* values are Bonferroni adjusted.

Abbreviation: BMI, body mass index.

Fasting glucose at <20, 24 to 28 and 35 to 37 weeks of gestation was associated with boys' sum of skinfolds, and at 24 to 28 and 35 to 37 weeks of gestation with girls' sum of skinfolds. Triglycerides at <20 weeks were positively associated with the sum of skinfolds in girls, and 35 to 37 weeks with sum of skinfolds of boys. Fatty acids were associated with sum of skinfolds in girls at <20 and 24 to 28 weeks. Gestational weight gain at <20 weeks was positively associated with boys' sum of skinfolds (Table 2). Table S2 describes the associations of maternal parameters that were included in the factors with neonatal adiposity.

## 4 DISCUSSION

**Figure 2:**
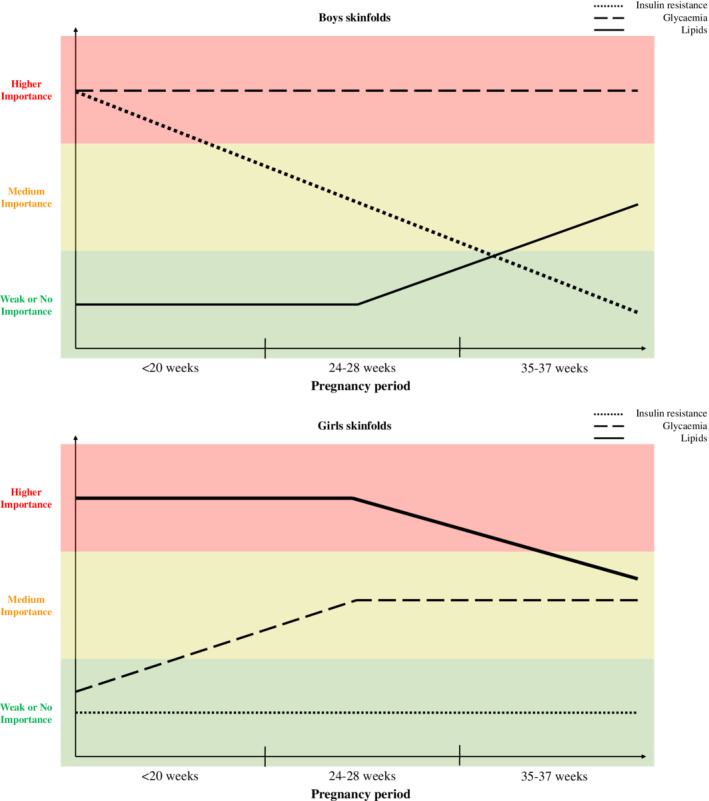
Summarized visualization of the degree of importance of some maternal parameters in relation to neonatal sum of skinfolds by sex

The main aim of the present study was to identify the effect of some maternal metabolism on neonatal skinfolds at different times in pregnancy in a sex‐dependent manner. Results confirmed our hypotheses that associations of metabolic parameters with neonatal adiposity change between different time‐periods and differ between sexes. We demonstrate a complex pattern of metabolic metabolism and its association with neonatal fat measured as sum of skinfolds, as illustrated in Figure 2. In summary, insulin resistance in early pregnancy is an important predictor for boys' sum of skinfolds, in addition to fasting glucose throughout pregnancy. In girls, maternal lipids in the first half of pregnancy period play a role for sum of skinfolds, as well as fasting glucose in the second half of pregnancy.

In both sexes, the early pregnancy period is relevant for neonatal adiposity, although for different metabolic parameters (ie, insulin resistance in boys and lipid levels in girls). Although a later intervention was effective in reducing neonatal adiposity,^22^ our data suggest that ideally maternal metabolism shall be normalized already early in pregnancy to reduce the risk of neonatal adiposity. Hence, interventions beginning prior to or in very early pregnancy might be even more effective than those initiated later, and achieved a 9% reduction in neonatal fat.^22^ Our results also indicate that both types of nutrients, glucose and lipids, are related to neonatal adiposity, although associations are time‐ and sex‐dependent. This argues for future time‐ and sex‐specific studies to delineate metabolic pathways between maternal adiposity, insulin resistance, the different nutrients and neonatal adiposity.

The physiological pathways influencing neonatal adiposity originate from maternal insulin resistance, which is higher with increasing maternal BMI. Indeed, in the Healthy Start Study^7^ maternal insulin resistance in the first half of pregnancy was an independent predictor of neonatal adiposity. This is in principle in line with our findings of insulin resistance (represented by factor 1) in early pregnancy playing a role, but in our study, this was limited to boys only. Later in pregnancy, the positive association of insulin resistance with neonatal adiposity was found in both boys and girls. The strong determining role of early insulin resistance for neonatal adiposity is not without precedent and extends to the pre‐pregnancy period, in which insulin resistance had the strongest association with neonatal fat mass, more so than insulin resistance in late pregnancy.^23^ However, neither of those previous studies tested for sex differences in this association or, specifically, in pregnant women with obesity. Two studies that analyzed boys and girls separately^24^, ^25^ found associations of insulin resistance late in pregnancy with measures of adiposity in girls, but not in boys, which is not fully in line with our findings. The different finding might be explained by differences in maternal BMI or the participants' level of glucose tolerance, since those other studies were not limited to women with obesity without GDM at baseline, as in our study sample.^24^, ^25^

The pathway from insulin resistance to neonatal adiposity might be through one of the nutrients glucose or lipids.^8^ Although found in various populations,^7^, ^24^, ^25^, ^26^ the role of fasting glucose for neonatal adiposity in women with obesity is a novel finding to the best of our knowledge. Maternal fasting glucose was a driver of fat accretion throughout pregnancy in boys and mainly in later pregnancy in girls. The sex difference in early pregnancy might explain why the Healthy Start Study^7^ only found an association of fasting glucose after 20 weeks of gestation, but not before, with neonatal adiposity. The reasons for the sex‐difference in the timing of the association of fasting glucose with neonatal fat remain speculative. The glucose steal phenomenon posits foetal hyperinsulinemia, the main driver of fat accretion, as the result of maternal hyperglycemia from early on.^27^ A sex‐difference in the timing of the glucose steal may be one explanation for our findings, but this remains to be studied.

Interestingly, independent of maternal insulin sensitivity, lipids in the first half of the pregnancy period were associated with sum of skinfolds in girls. In boys, only triglycerides at 35 to 37 weeks were related with neonatal skinfolds. This sex interaction in the association of lipids with neonatal skinfolds might explain the lack of association in previous studies not distinguishing between sexes.^7^, ^19^, ^20^ To complicate matters further, the association of maternal lipids with neonatal adiposity might also depend on maternal metabolic status and/or weight. Crume et al^7^ observed that maternal triglycerides, in middle to late pregnancy, were associated with neonatal fat mass only in women with obesity before pregnancy. In a study including women with a normal glucose metabolism and those with gestational diabetes, maternal lipids, triglycerides and free fatty acids in late pregnancy were positively associated with neonatal fat mass only in women with gestational diabetes, but not in women with normal glycaemia.^28^ This complex interaction between maternal phenotype, neonatal sex and maternal lipid profile could be a reason why in a Mendelian randomization approach no causal effect of maternal triglyceride concentrations on offspring birth weight was found.^29^ Mendelian randomization is a powerful method for establishing causal exposure‐outcome relations. The present results call for using this method in a sex‐specific manner, and at the same time distinguishing between women with different weight status. Moreover, neonatal fat percentage is the relevant offspring outcome more sensitive to in utero influences, and hence, more variable than birth weight.

While analysis of the insulin secretory response (factor 2) did not result in a clear picture, glucose tolerance (factor 3) at the end of pregnancy was related to neonatal adiposity for boys and girls. Conflicting results have been found on the association of glucose tolerance with neonatal adiposity. Most,^7^, ^25^, ^30^ but not all,^23^ studies reported positive associations. None of those assessed sex differences, and importantly, only one study evaluated exclusively pregnant women with obesity. Thus, more information is needed to draw any conclusions on the role of insulin secretory response or glucose tolerance for neonatal adiposity.

Maternal adiposity, mostly assessed by the pre‐pregnancy BMI, has been related with neonatal adiposity in numerous studies,^4^, ^5^, ^25^, ^31^, ^32^, ^33^ although one reported no association.^34^ We found maternal adiposity to be associated with neonatal adiposity in boys throughout pregnancy, but no association was found in girls. Although, when analyzed individually, maternal BMI in the second half of pregnancy period was associated with the girls' adiposity. Different from many of the previously mentioned studies, we only had women with obesity in our sample, which might have precluded finding strong associations with maternal adiposity. Reports on sex differences in the association of maternal adiposity and neonatal adiposity are conflicting: Some find associations only in boys,^35^ but other studies only in girls.^36^, ^37^ Prior studies in pregnant women with diabetes have reported an interaction of foetal sex with a number of maternal variables in the prediction of large or small‐for‐gestational age new‐borns but results are not comparable since the outcome variable was not new‐born adiposity and maternal variables did not include lipids or insulin sensitivity.^38^

### 4.1 Sex and time differences

Conceptually, male and female foetuses follow different growth strategies in utero.^39^, ^40^ Mechanisms are not well understood, but male embryos have more rapid cell divisions compared to females,^41^ which could lead to a more rapid growth.^39^, ^40^ Their higher growth rate might explain why male foetuses are also more responsive to changes in nutrient supply.^39^, ^40^ In addition, the peak growth velocity of males seems to be later in pregnancy compared to female foetuses.^39^ These different growth strategies might result in differences in nutritional needs, at different times in pregnancy. The results of our study provide strong evidence to support this concept. However, more systematic assessments of time‐ and sex‐dependent associations of maternal metabolism with neonatal adiposity are needed in future studies.

Although foetal fat is mostly accumulated late in pregnancy,^42^, ^43^ our data highlight that also the early pregnancy period is important for neonatal adiposity. This finding is in line with other studies, showing that maternal metabolism that led to gestational diabetes diagnosis later in pregnancy, had an influence on foetal abdominal circumference already at 17 weeks of pregnancy, with a more pronounced effect in males.^9^ Also maternal obesity was related to accelerated growth at 20 weeks of gestation,^10^ without sex differences studied.

### 4.2 Strengths and limitations

The European representativeness of our study sample with trials sites spread over Europe is a strength, but the restriction to women with obesity can be seen as a limitation of our study. However, since the prevalence of pregnant women with obesity is alarming,^44^ studying the consequences of maternal obesity is of public health relevance. A strength of our study is the measurements of maternal metabolic parameters at three time points in pregnancy. Without this elaborate approach, identification of time‐dependent effects had not been possible.

DALI study was designed to evaluate the effects of different interventions on preventing gestational diabetes in pregnant women with obesity. Although we adjusted the analyses for the various interventions, we cannot fully exclude a minor influence on the results. While more direct measures of neonatal adiposity by for example, air displacement plethysmography, would have been preferred, we recently reported an association of sum of skinfolds with cord blood leptin in this cohort,^22^ supporting the validity of the skinfold measures as proxy for neonatal adiposity.

## 5 CONCLUSION

Our results show time‐dependent associations of maternal metabolic parameters with neonatal adiposity. Importantly, and a novel key result, we demonstrate that this time‐dependence varies between sexes. Thus, the study highlights the urgent need for inclusion of neonatal sex in all analyses, not as confounder, but as major determinant and modifying factor. This has been recommended previously.^45^, ^46^ The present study can inform future studies to include larger sample sizes, because of the sex differences, but also measurements of maternal metabolism at more than one time point during pregnancy.

## CONFLICT OF INTEREST

No conflict of interest was declared.

## AUTHOR CONTRIBUTIONS

R.L. designed this study, conducted the statistical analysis, contributed to the interpretation of the results and drafted the manuscript. G.D. and M.v.P. designed this study, contributed to the interpretation of the results and drafted the manuscript. All authors, except R.L., made substantial contribution to the conception of the DALI study and acquisition of data. All authors revised the manuscript and contributed to the content. All authors approved the final manuscript as submitted and agree to be accountable for all aspects of the work.

## Supporting information


**Table S1** Cumulative explained variance of each factor in three periods of gestation.

**Table S2** Multilevel regression coefficients of the association between maternal health profile components and child's sum of skinfolds (mm) by sex in three periods of gestation.
Legend: Adjusted for intervention group, site of recruitment, maternal ethnicity, education, BMI (except when BMI was the exposure), age and smoking status, gestational age during pregnancy (weeks), gestational age at birth (weeks) and neonatal age at measurement (hours), and cluster structure (individuals nested in site of measurement). Besides the aforementioned adjustments, HOMA‐index was also an adjustment when triglycerides and fatty acids were the exposures. p values are Bonferroni adjusted
