# Supplementary material for: Temporal relationships between maternal metabolic parameters with neonatal adiposity in women with obesity differ by neonatal sex: Secondary analysis of the DALI study
Source: Pediatr Obes. 2020 Mar 6;15(7):e12628. doi: 10.1111/ijpo.12628 (PMC7317347; doi:10.1111/ijpo.12628)
Supplement: Supplementary file 1 — Table S1 Cumulative explained variance of each factor in three periods of gestation. [file IJPO-15-e12628-s001.docx]

**Supplementary Table 1.** Cumulative explained variance of each factor in three periods of gestation.

| Cumulative explained variance | | | |
| --- | --- | --- | --- |
| Factors | Weeks of gestation | | |
|  | <20 weeks | 24-28 weeks | 35-37 weeks |
| Factor 1  Fasting insulin and HOMA–IR | 0.36 | 0.38 | 0.34 |
| Factor 2 One-hour and two-hour insulin,  and Stumvoll 1^st^ and 2^nd^ phases | 0.63 | 0.59 | 0.60 |
| Factor 3 One-hour and two-hour glucose | 0.77 | 0.72 | 0.72 |
| Factor 4 Leptin, BMI and neck circumference | 0.89 | 0.82 | 0.84 |
