# Supplementary material for: Temporal relationships between maternal metabolic parameters with neonatal adiposity in women with obesity differ by neonatal sex: Secondary analysis of the DALI study
Source: Pediatr Obes. 2020 Mar 6;15(7):e12628. doi: 10.1111/ijpo.12628 (PMC7317347; doi:10.1111/ijpo.12628)
Supplement: Supplementary file 2 — Table S2 Multilevel regression coefficients of the association between maternal health profile components and child's sum of skinfolds (mm) by sex in three periods of gestation. Legend: Adjusted for intervention group, site of recruitment, maternal ethnicity, education, BMI (except when BMI was the exposure), age and smoking status, gestational age during pregnancy (weeks), gestational age at birth (weeks) and neonatal age at measurement (hours), and cluster structure (individuals nested in site of measurement). Besides the aforementioned adjustments, HOMA‐index was also an adjustment when triglycerides and fatty acids were the exposures. p values are Bonferroni adjusted [file IJPO-15-e12628-s002.docx]

**Supplementary Table 2.** Multilevel regression coefficients of the association between maternal health profile components and child’s sum of skinfolds (mm) by sex in three periods of gestation.

| Maternal parameters | Boys | | | | | | Girls | | | | | |
| --- | --- | --- | --- | --- | --- | --- | --- | --- | --- | --- | --- | --- |
|  | <20 weeks | | 24-28 weeks | | 35-37 weeks | | <20 weeks | | 24-28 weeks | | 35-37 weeks | |
|  | β | p | β | p | β | p | β | p | β | p | β | p |
| One-hour Glucose (mmol/l) | -0.156 | 0.486 | -0.104 | 0.517 | 0.272 | 0.190 | 0.190 | 0.448 | 0.433 | 0.087 | 0.322 | 0.223 |
| Two-hour Glucose (mmol/l) | 0.509 | **0.011** | -0.090 | 0.767 | 0.525 | **0.008** | 0.269 | 0.290 | 0.717 | **0.011** | 0.807 | **<0.001** |
| Fasting Insulin (log) | 1.551 | **0.005** | 0.173 | 0.822 | 1.163 | **0.017** | 0.939 | **0.015** | 0.475 | 0.582 | 1.312 | **0.044** |
| One-hour Insulin (log) | 0.123 | 0.748 | 1.231 | 0.113 | 1.434 | 0.059 | 0.123 | 0.908 | 0.803 | 0.308 | 0.233 | 0.719 |
| Two-hour Insulin (log) | 0.633 | **0.044** | 0.566 | 0.379 | 1.407 | **<0.001** | -0.298 | 0.361 | 1.172 | **0.023** | 0.702 | 0.209 |
| HOMA-IR (log) | 1.587 | **0.003** | 0.331 | 0.622 | 1.240 | **0.013** | 0.824 | 0.056 | 0.775 | 0.319 | 1.292 | **0.033** |
| Stumvoll 1st phase (log) | 1.318 | 0.161 | 0.781 | 0.270 | 1.528 | **0.032** | 1.323 | 0.103 | 1.311 | 0.245 | 0.952 | 0.278 |
| Stumvoll 2nd phase (log) | 1.332 | 0.170 | 0.812 | 0.267 | 1.583 | **0.032** | 1.354 | 0.114 | 1.427 | 0.235 | 0.974 | 0.278 |
| Leptin (log) | 0.485 | 0.526 | 0.087 | 0.898 | 0.017 | 0.983 | 0.371 | 0.503 | -0.162 | 0.839 | -0.148 | 0.768 |
| Neck circumference (cm) | 0.519 | **0.005** | 0.316 | 0.156 | 0.246 | 0.163 | 0.148 | 0.480 | -0.019 | 0.926 | -0.022 | 0.913 |
| BMI (log) | 5.560 | **0.010** | 5.962 | **0.022** | 8.032 | **<0.001** | 2.216 | 0.136 | 3.278 | **0.036** | 3.968 | **0.019** |

Legend: Adjusted for intervention group, site of recruitment, maternal ethnicity, education, BMI (except when BMI was the exposure), age and smoking status, gestational age during pregnancy (weeks), gestational age at birth (weeks) and neonatal age at measurement (hours), and cluster structure (individuals nested in site of measurement). Besides the aforementioned adjustments, HOMA-Index was also an adjustment when triglycerides and fatty acids were the exposures. p values are Bonferroni adjusted
